# Peri-Operative Surgical Risk and Postoperative Outcomes in Patients With COPD Undergoing Abdominal Surgery: A Retrospective Observational Study

**DOI:** 10.7759/cureus.105280

**Published:** 2026-03-15

**Authors:** Rajeebshankar Karmakar, Ashraf Hossain, Md S Tahmid, Dipannita Biswas, Md F Kamal, Sushit K Biswas, Suman Kundu, Sutopa Halder Supti, Tumpa Kundu, Israt Jahan, Masnoon A Noor

**Affiliations:** 1 Thoracic Surgery, Faridpur Medical College and Hospital, Faridpur, BGD; 2 Surgery, Mymensingh Medical College Hospital, Mymensingh, BGD; 3 Trauma and Orthopaedics, Mymensingh Medical College Hospital, Mymensingh, BGD; 4 Trauma and Orthopaedics, Sher-e-Bangla Medical College Hospital, Barisal, BGD; 5 General Surgery, Bangladesh Medical University, Dhaka, BGD; 6 General Surgery, Faridpur Medical College Hospital, Faridpur, BGD; 7 Critical Care Medicine, Kuwait Bangladesh Friendship General Hospital, Dhaka, BGD; 8 Paediatric Gastroenterology, Bangabandhu Sheikh Mujib Medical University (BSMMU), Dhaka, BGD; 9 Rheumatology, Bangladesh Medical University, Dhaka, BGD; 10 Public Health, Institute of Research, Innovation, Incubation and Commercialization (IRIIC), Dhaka, BGD; 11 Thoracic Surgery, Bangabandhu Sheikh Mujib Medical University (BSMMU), Dhaka, BGD

**Keywords:** chronic obstructive pulmonary disease, copd, copd mortality, obstructive airway disease, peri-operative surgical risk

## Abstract

Background

Chronic obstructive pulmonary disease (COPD) is a major global health burden and a leading cause of death and disability, commonly encountered in surgical populations where it independently predicts adverse outcomes. Increased postoperative mortality and morbidity arise from pathophysiological changes such as lung hyperinflation, intrinsic positive end-expiratory pressure (PEEP), impaired gas exchange, and reduced lung volumes after major abdominal surgery. The interaction between disease severity, surgical factors, and anesthesia duration underscores the need for structured peri- and postoperative risk assessment to reduce complications in this vulnerable group.

Methods

This retrospective observational study, conducted over 23 months at Faridpur Medical College Hospital, reviewed the records of 107 consecutive patients aged 40 years and old with spirometrically confirmed COPD who underwent elective abdominal surgery under general anesthesia. Strict inclusion and exclusion criteria were applied to ensure diagnostic accuracy and minimize bias, focusing on patients with post-bronchodilator first second of forced expiration (FEV_1_) to the full, forced vital capacity (FVC) (FEV₁/FVC) <0.7 and American Society of Anesthesiologists (ASA) class up to IV while excluding emergency, trauma, transplant, and incomplete cases. Comprehensive peri-operative data, including demographic characteristics, pulmonary function indices, laboratory parameters, intra-operative variables, and postoperative complications and outcomes, were systematically extracted to evaluate surgical risk in this vulnerable population.

Results

The cohort was elderly (70.06±8.49 years), predominantly men (81, 75.7%), with high smoking exposure (70, 65.4%) and hypertension (68, 63.5%), alongside diabetes (25, 23.4%) and heart disease (31, 28.9%); mean BMI was 28.14±3.4 kg/m² with preserved albumin 4.1±0.7 g/dL and creatinine 0.8±0.3 mg/dL. Most patients had a higher anesthetic risk, with 48 (44.9%) in ASA III and 20 (18.7%) in ASA IV, while spirometry showed preserved FEV₁ (92.4±14.4%) and FVC (103.7±14.2%) but reduced FEV₁/FVC (60.9±6.3%), confirming obstruction. Cholecystectomy (26, 24.3%) and appendectomy (22, 20.6%) predominated, with a mean operation time of 190.5±47.6 minutes and anesthesia time of 220.7±31.9 minutes. Postoperative pulmonary complications were notable, particularly atelectasis (10, 9.3%), pneumonia (9, 8.4%), refractory hypoxia (9, 8.4%), and prolonged ventilation (8, 7.5%), while arrhythmia (17, 15.9%) was the most frequent cardiac event. ICU stay was 8.7±3.2 days and hospital stay 18.2±5.3 days, with overall morbidity (16, 14.9%) and mortality (2, 1.9%).

Conclusion

Patients with COPD undergoing abdominal surgery represent a high-risk population characterized by advanced age, multiple comorbidities, and significant peri-operative vulnerability, where preserved spirometry does not eliminate the risk of pulmonary and cardiac complications. The relatively low mortality observed suggests that structured pre-operative evaluation, optimized intra-operative care, and vigilant postoperative monitoring can effectively reduce adverse outcomes in this complex group.

## Introduction

Chronic obstructive airway diseases, particularly chronic pulmonary obstructive disease (COPD), were the fourth leading cause of death globally in 2008 and are projected to become the third leading cause by 2020-2030, while also ranking ninth in years of life lost according to WHO data [1,2]. Within surgical populations, COPD is common, present in approximately 5%-10% of patients undergoing abdominal surgery, and is an independent predictor of adverse postoperative outcomes [3,4]. Patients with COPD experience markedly higher 30-day mortality (6.7% vs 1.4%) and morbidity (25.8% vs 10.2%) compared with non-COPD patients [1,5]. These adverse outcomes are driven by profound pathophysiological changes, including static and dynamic lung hyperinflation, intrinsic positive end-expiratory pressure (PEEP) reaching 6-9 cmH₂O, and postoperative reductions in vital capacity of up to 70% following upper abdominal surgery [6-8]. Functional residual capacity declines within 16-20 hours after surgery, often falling below closing volume, leading to atelectasis, ventilation-perfusion mismatch, and hypoxemia [6]. The mechanical work of breathing may increase up to tenfold at rest in severe COPD, compounded by diaphragmatic dysfunction, low BMI, and impaired cough, which heighten susceptibility to residual neuromuscular blockade and postoperative pneumonia [8-10]. Surgical and anesthetic factors further amplify risk, as upper abdominal and thoracic surgeries are associated with pulmonary complication rates of 19.7%-56%, compared with 7.7% in lower abdominal procedures, while complication incidence rises from 4% for operations under one hour to 73% for procedures exceeding four hours [11-12]. Beyond pulmonary events, COPD significantly increases the risk of sepsis, septic shock, acute kidney injury, myocardial infarction, cardiac arrest, and prolonged ICU and hospital stay [1,13]. Given the high prevalence of COPD, its distinct pathophysiology, and the strong interaction between patient factors, surgery type, anesthesia duration, and postoperative complications, systematic evaluation of peri- and post-operative risk in this population is essential to improve surgical outcomes and reduce morbidity and mortality. This study aimed to evaluate the peri-operative risk factors and postoperative outcomes in patients with spirometry-confirmed COPD undergoing abdominal surgery.

## Materials and methods

This retrospective observational study was conducted in the Department of Surgery of Faridpur Medical College Hospital over a 23-month period from January 2024 to November 2025, aiming to evaluate peri- and post-operative risk among patients with COPD undergoing abdominal surgery. Hospital records were systematically reviewed, and a total of 107 patients were included using a consecutive sampling technique, whereby all eligible cases within the study period were enrolled to minimize selection bias and ensure comprehensive case capture. The study population comprised patients aged ≥40 years with spirometrically confirmed COPD, defined by a post-bronchodilator first second of forced expiration (FEV1) to the full, forced vital capacity (FVC) (FEV₁/FVC) ratio of <0.7, who underwent common abdominal operations under general anesthesia, including procedures such as cholecystectomy, appendectomy, exploratory laparotomy or laparoscopy, and other gastrointestinal surgeries. Only patients with American Society of Anesthesiologists (ASA) physical status classifications up to Class IV were considered eligible to allow the inclusion of individuals with significant systemic disease while excluding moribund cases. Patients were excluded if the diagnosis of COPD was unconfirmed or based solely on suggestive symptoms without spirometric validation, if they were trauma or transplant cases, if aged <16 years, if the surgery was performed on an emergency basis, if they required ICU admission prior to surgery, or if their records contained incomplete or missing essential data. Demographic variables such as age, sex, body mass index, smoking history, and comorbidities were extracted, alongside pre-operative laboratory parameters, including serum albumin and creatinine, and pulmonary function indices such as FEV₁ (% predicted), FVC (% predicted), and FEV₁/FVC ratio. Intra-operative variables, including the type of operation, operation time, and anesthesia time, were recorded, as well as postoperative outcomes such as ICU stay, hospital stay, pulmonary, cardiac, renal, infectious, and thrombotic complications, overall morbidity, and in-hospital mortality. Data was compiled using a structured data collection sheet. Statistical analysis was performed using IBM SPSS Statistics, version 29 (IBM Corp., Armonk, NY). Continuous variables were expressed as mean±SD and categorical variables as frequency and percentage.

## Results

Table 1 presents the demographic and baseline clinical characteristics of the study population, revealing a predominantly elderly cohort with a mean age of 70.06±8.49 years, and largely men (81, 75.7%) with 26 women (24.3%), reflecting a marked gender imbalance in this COPD surgical cohort. The mean BMI was 28.14±3.4 kg/m², and a substantial proportion had a history of smoking (70, 65.4%), underscoring the chronic exposure profile typical of obstructive airway disease. Comorbid conditions were highly prevalent, particularly hypertension (68, 63.5%), followed by heart disease (31, 28.9%) and diabetes mellitus (25, 23.4%), while renal disease (6, 5.6%), malignancy (5, 4.7%), and liver disease (3, 2.8%) were less frequent. Despite this considerable systemic burden, biochemical parameters suggested relatively preserved nutritional and renal status, with serum albumin levels of 4.1±0.7 g/dL and serum creatinine at 0.8±0.3 mg/dL.

**Table 1:** Distribution of respondents according to demographic variables.

Variables	Value
Age (mean±SD)	70.06 ± 8.49 years
Sex	
Male (n,%)	81 (75.7%)
Female (n,%)	26 (24.3%)
Body mass index (mean±SD)	28.14±3.4 kg/m²
Smoking history (n,%)	70 (65.4%)
Comorbidities	
Hypertension (n,%)	68 (63.5%)
Diabetes mellitus (n,%)	25 (23.4%)
Liver disease (n,%)	3 (2.8%)
Heart disease (n,%)	31 (28.9%)
Renal disease (n,%)	6 (5.6%)
Malignancy (n,%)	5 (4.7%)
Serum albumin (mean±SD)	4.1±0.7 g/dL
Serum creatinine (mean±SD)	0.8±0.3 mg/dL

Table 2 illustrates the distribution of the study population according to the ASA physical status classification and shows that the majority of patients were clustered within the higher ASA categories, reflecting significant systemic disease burden at the time of surgery. Thirty-nine (36.4%) patients were classified as ASA Class II. A substantial proportion fell into ASA Class III (48, 44.9%), and 20 (18.7%) were categorized as Class IV, indicating severe systemic illness with potential life-threatening implications.

**Table 2:** Distribution of respondents according to ASA classification.

ASA classification	Value (n,%)
Class I	0 (0%)
Class II	39 (36.4%)
Class III	48 (44.9%)
Class IV	20 (18.7%)

Table 3 demonstrates the pulmonary function profile of the study population and reflects a characteristic obstructive pattern with relatively preserved lung volumes. The mean FEV₁ was 92.4±14.4% predicted and the mean FVC was 103.7±14.2% predicted, indicating a maintained expiratory capacity and vital capacity in most patients. However, the mean FEV₁/FVC ratio was reduced to 60.9±6.3%, confirming airflow limitation consistent with chronic obstructive pulmonary disease. This combination of preserved volumes with a decreased ratio suggests mild to moderate obstruction rather than advanced ventilatory failure.

**Table 3:** Distribution of respondents according to intra operative variables.

Variables	No. of patiens, n (%)
Type of operation	
Exploratory laparotomy/aparoscopy	16 (14.9%)
Cholecystectomy	26 (24.3%)
Gastrectomy	4 (3.7%)
Gastrectomy with gastrojejunostomy	2 (1.9%)
Colectomy	1 (0.9%)
Colonic anastomosis and ileostomy	1 (0.9%)
Ileostomy	5 (4.7%)
Small bowel resection	2 (1.9%)
Appendectomy	22 (20.6%)
Pancreatic resection	1 (0.9%)
Orthopedic surgery	15 (14%)
Head and neck surgery (n,%)	4 (3.7%)
Hernia repair (n,%)	8 (7.5%)
Operation time (mean±SD)	190.5±47.6 minutes
Anesthesia time (mean±SD)	220.7±31.9 minutes

Table 4 outlines the intra-operative characteristics of the study population, reflecting common abdominal procedures, with cholecystectomy (26, 24.3%) and appendectomy (22, 20.6%) being the most frequent operations, followed by exploratory laparotomy/laparoscopy (16, 14.9%) and orthopedic surgery (15, 14%). Less common procedures included hernia repair (8, 7.5%), ileostomy (5, 4.7%), gastrectomy (4, 3.7%), head and neck surgery (4, 3.7%), small bowel resection (2, 1.9%), gastrectomy with gastrojejunostomy (2, 1.9%), and isolated cases of colectomy (1, 0.9%), colonic anastomosis with ileostomy (1, 0.9%), and pancreatic resection (1, 0.9%). The mean operation time was 190.5±47.6 minutes, while the mean anesthesia time extended to 220.7±31.9 minutes, reflecting prolonged peri-operative exposure.

**Table 4:** Distribution of respondents according to pulmonary function test. FEV_1_: first second of forced expiration to the full; FVC: forced vital capacity.

Lung function test	Result (mean±SD)
FEV_1_ (% Predicted)	92.4 ± 14.4
FVC (% Predicted)	103.7 ± 14.2
FEV_1_/FVC (% Predicted)	60.9 ± 6.3

Table 5 presents the spectrum of postoperative complications observed in the study population and reflects a clinically significant burden of adverse outcomes following surgery in patients with COPD. Pulmonary complications constituted a substantial proportion of events, with atelectasis (10, 9.3%) being the most frequent event, followed by pneumonia (9, 8.4%), refractory hypoxia (9, 8.4%), and prolonged mechanical ventilation (8, 7.5%). Additional respiratory events included pleural effusion (6, 5.6%), respiratory failure (5, 4.7%), and isolated cases of empyema (1, 0.9%) and re-intubation (1, 0.9%). Among cardiac complications, arrhythmia was the most common (17, 15.9%), while cardiac arrest (1, 0.9%) and myocardial infarction (1, 0.9%) were rare but serious occurrences. Renal complications were infrequent, including acute kidney injury (2, 1.9%) and dialysis requirement (2, 1.9%), with acute urinary retention noted in three (2.8%) patients. Infectious events included infection (8, 7.5%), wound infection (7, 6.5%), sepsis (4, 3.7%), and septic shock (2, 1.9%), while deep vein thrombosis occurred in two (1.9%) patients, collectively reflecting a measurable yet varied postoperative morbidity burden in this COPD surgical cohort.

**Table 5:** Distribution of respondents according to post operative complications.

Complications	Prevalence, n (%)
Pulmonary complications	
Prolonged mechanical ventilation	8 (7.5%)
Pneumonia	9 (8.4%)
Refractory hypoxia	9 (8.4%)
Pleural effusion	6 (5.6%)
Atelectasis	10 (9.3%)
Respiratory failure	5 (4.7%)
Empyema	1 (0.9%)
Re-intubation	1 (0.9%)
Cardiac complications	
Arrhythmia	17 (15.9%)
Cardiac arrest	1 (0.9%)
Myocardial infarction	1 (0.9%)
Renal complications	
Acute kidney injury	2 (1.9%)
Dialysis requirement	2 (1.9%)
Acute urinary retention	3 (2.8%)
Infectious and thrombotic complications	
Infection	8 (7.5%)
Sepsis	4 (3.7%)
Septic shock	2 (1.9%)
Wound infection	7 (6.5%)
Deep vein thrombosis	2 (1.9%)

Table 6 summarizes the postoperative outcomes of the study population and reflects a moderate requirement for critical care support, with a mean ICU stay of 8.7±3.2 days, followed by a prolonged overall hospital stay of 18.2±5.3 days, reflecting the recovery demands in this elderly COPD cohort. Overall postoperative morbidity was observed in 16 (14.9%) patients, indicating a measurable burden of complications despite structured peri-operative care. In-hospital mortality occurred in two (1.9%) patients, suggesting that fatal outcomes were relatively infrequent. Together, these findings portray a population with significant peri-operative vulnerability yet generally controlled short-term outcomes under institutional management.

**Table 6:** Distribution of respondents according to postoperative outcomes.

Outcomes	Result
ICU stay (mean±SD)	8.7±3.2 days
Hospital stay (mean±SD)	18.2±5.3 days
Morbidity	16 (14.9%)
Mortality	2 (1.9%)

## Discussion

Our study explored peri-operative risk and postoperative outcomes among patients with COPD undergoing abdominal surgery in a real-world clinical setting. Our findings reveal a complex interplay between advanced age, systemic comorbidities, anesthetic exposure, and respiratory physiology in shaping surgical outcomes. In this discussion, our results are interpreted in light of the existing literature to better understand their clinical implications and relevance to peri-operative practice. Our cohort portrays an elderly, predominantly male COPD surgical population with frequent smoking exposure and a heavy cardiometabolic comorbidity burden, while baseline nutritional and renal biochemical markers remain generally preserved. A comparable age profile was reported elsewhere, with a median age of 70.5 years in one study and a mean age of 70.6±8.3 years in another [9,14]. Male predominance was similarly marked in prior cohorts, with male:female distributions of 83.5:16.5 and 84.3:15.7 [8,14]. In contrast to our relatively higher BMI, lower mean BMI values were reported in other series, such as 23.33±2.98 kg/m² and 23.0±3.1 kg/m², suggesting potential regional or case-mix differences in nutritional phenotype [2,9]. Smoking exposure in published cohorts varied widely, with one study reporting 74.8%, while another reported only 34%, reflecting heterogeneity in smoking epidemiology and population selection [2,3]. Hypertension prevalence in comparator studies ranged from 73.5% to 53.7%, broadly consistent with a high baseline cardiovascular risk [1,15]. Diabetes prevalence in other cohorts was also similar in scale, reported as 20.5% and 21.2% [9,13]. Liver disease was uncommon across studies, reported as 2.84% and 2.8%, echoing the low burden seen in our population [1,13]. Cardiac disease prevalence in the literature clustered around our pattern, with 30% reported in one cohort and 32.8% in another [3,12]. Renal disease similarly remained low but present, reported as 4.9% and 3.5% [2,9]. A striking difference emerged for malignancy, which was highly prevalent in oncologic-dominant cohorts, 71.6% in one report and 25% in another, whereas our cohort appeared more representative of mixed non-oncologic general surgery [8,11]. Baseline laboratory values were comparable to prior reports, with serum albumin around 4.3 g/dL and creatinine around 0.9 mg/dL in one study, aligning with the preserved biochemical profile observed in our patients [8].

Our study population demonstrates a clear predominance of patients within the higher ASA categories, reflecting a population with moderate to severe systemic disease and relatively few individuals in the lowest anesthetic risk group. In comparison, one study reported Class II in 50.0%, Class III in 34.4%, and Class IV in 15.6%, suggesting a stronger clustering within intermediate risk strata rather than advanced systemic compromise [11]. Another study showed a markedly different distribution, with Class I accounting for 11.4%, Class II for 80.2%, and Class III for only 8.4%, indicating that the majority of their surgical patients belonged to lower anesthetic risk categories [2]. Compared with these findings, our population appears to carry a relatively heavier burden of systemic illness, as reflected by the higher concentration of patients in ASA Class III and IV, thereby suggesting a more medically complex and potentially higher-risk surgical cohort.

Patients of our study demonstrated relatively preserved expiratory volumes with a reduced FEV₁/FVC ratio, reflecting an obstructive ventilatory pattern without profound impairment of lung volumes. A similar physiological profile was reported in one study where FEV₁ was 94% predicted and FVC was 103.64±15.86% predicted, indicating preserved vital capacity despite airflow limitation [2]. Another investigation documented FEV₁ of 84.5±21.1% predicted and FVC of 104.1±20.5% predicted, again suggesting maintained lung volumes with obstructive characteristics [14]. Compared with these reports, our findings align closely with cohorts demonstrating mild to moderate airflow obstruction rather than advanced ventilatory compromise, underscoring that even patients with relatively preserved spirometric indices may remain vulnerable in the peri-operative setting.

Our operative profile reflects a broad general-surgery case mix dominated by common abdominal procedures, with fewer major resections and a moderate representation of non-abdominal operations, alongside operative and anesthetic durations that suggest meaningful intra-operative exposure in a COPD population. A higher proportion of exploratory laparotomy/laparoscopy was reported elsewhere, with 40.6% in one cohort and 33.2% in another, indicating a stronger weighting toward exploratory major abdominal surgery than in our series [8,11]. Lower rates of cholecystectomy (9.3%) and appendectomy (3.12%) were described in one study, while the same cohort reported higher proportions of gastrectomy (9.37%), gastrectomy with gastrojejunostomy (3.12%), colectomy (3.12%), colonic anastomosis with ileostomy (3.12%), small bowel resection (9.3%), pancreatic resection (3.12%), and ileostomy (6.25%), suggesting a relatively more “major abdominal” operative burden compared with our predominantly routine abdominal surgery pattern [11]. For orthopedic procedures, one study reported 5.99% while another reported 12.8%, broadly aligning with the modest presence of non-abdominal surgery seen in our cohort [1,2]. Head-and-neck surgery showed marked variability across the literature, with 34.8% in one cohort and 11.6% in another, whereas our series contained relatively fewer such cases, reflecting differences in institutional surgical case-mix [2,14]. Hernia repair also varied, reported as 11.17% in one study and 4.7% in another, consistent with the heterogeneity of “low-to-moderate complexity” operations across COPD surgical cohorts [1,12]. Duration metrics further emphasize this variability; one study reported a mean operation time of 393±149 minutes and another reported a median operation time of 221.3 minutes, both suggesting longer operative exposure than typically seen in our cohort [9,14]. Anesthesia duration ranged from 165 minutes in one report to 4.62 hours in another, reinforcing that differences in procedure complexity and peri-operative pathways substantially shape anesthetic exposure in COPD populations [1,2].

Our cohort exhibited a complication pattern dominated by postoperative pulmonary events and notable cardiac rhythm disturbance, while severe cardiopulmonary collapse, advanced renal failure, and thrombotic complications were relatively uncommon. For prolonged mechanical ventilation, rates in prior cohorts ranged from 9.4% in one study to 6% in another, broadly comparable to a moderate-risk postoperative ventilatory burden [11,12]. Pneumonia rates in published series were similarly variable, reported as 6.51% in one dataset and 7.9% in another, consistent with a persistent risk of infectious pulmonary complications in COPD surgical patients [1,14]. A comparable rate of refractory hypoxia was reported at 9.4% in one cohort, underscoring a shared vulnerability to postoperative oxygenation failure [11]. Pleural effusion displayed wide heterogeneity across studies, reaching 20.2% in one report but only 2.2% in another, suggesting that surgical mix, peri-operative fluid balance, and baseline cardiopulmonary reserve may strongly shape this outcome [2,8]. Atelectasis rates ranged from 7.9% in one cohort to 3.5% in another, highlighting the expected but variable burden of postoperative lung volume loss and secretion retention in obstructive disease [9,14]. Respiratory failure similarly varied, with 5.6% reported in one study and 9.4% in another, consistent with intermediate-risk respiratory decompensation across heterogeneous surgical settings [11,12]. Empyema remained rare, with one study reporting 0.9%, mirroring the low-frequency but clinically serious nature of this complication [9]. Re-intubation rates differed markedly, spanning 5.51% in one dataset to 0.24% in another, reflecting differences in airway management, postoperative monitoring, and thresholds for ventilatory rescue [1,14]. For cardiac complications, arrhythmia showed the greatest variability, reported as 21.9% in one cohort but only 1% in another, suggesting major differences in baseline cardiovascular risk and peri-operative surveillance intensity [2,11]. Catastrophic cardiac events were uncommon across studies, with cardiac arrest reported at 1.57% in one analysis and myocardial infarction reported at 0.8% and 0.57% in two separate datasets [1,13]. Renal events were generally infrequent, with acute kidney injury reported as 2.8% in one cohort and 1.5% in another, and dialysis requirement reported as 1.73% versus 0.5%, aligning with a low but meaningful multi-organ complication burden in COPD surgery [1,2,14]. Postoperative urinary retention was reported at 1.7% in one study, reinforcing its role as a smaller but relevant peri-operative morbidity marker [2]. Infectious outcomes remained variable, with overall infection reported as 15% in one cohort and 7.9% in another, while sepsis ranged from 11% to as low as 0.2%, and septic shock ranged from 5.01% to 0.2%, highlighting substantial differences in case severity, definitions, and peri-operative infection control practices [1,2,9,13,14]. Wound infection also varied, with 0.6% in one dataset and 6.7% in another, suggesting institutional and procedural influences on surgical-site risk [2,13]. Thrombotic events were inconsistently reported, with DVT documented as 1.67% in one study and 5.7% in another, supporting the concept that venous thromboembolism risk may be strongly shaped by prophylaxis practices, mobility, and patient selection [1,3].

Our findings reflect a postoperative course characterized by a moderate duration of critical care and hospitalization, with a measurable but relatively controlled complication burden and low short-term mortality. In comparison, one study reported a mean ICU stay of 14.5±19.7 days, while another documented a mean of 18.2 days, indicating substantially longer critical care utilization in certain COPD surgical cohorts [3,13]. Hospital stay in other series was shorter, reported as 7.4±11.8 days in one analysis and 8.7 days in another, suggesting that length of hospitalization may vary considerably depending on surgical complexity and peri-operative pathways [1,9]. Postoperative morbidity has been reported as 28.8% in one cohort and 17.3% in another, both exceeding the overall complication burden observed in our population [2,8]. Mortality rates of 6.7% in one study and 2.6% in another indicate higher short-term fatality compared with our experience, underscoring that outcomes in COPD surgical patients are highly influenced by patient selection, disease severity, and peri-operative management strategies [1,13].

Despite providing meaningful insights into peri-operative risk among patients with COPD undergoing abdominal surgery, this study has several limitations that merit consideration. The retrospective observational design inherently limits causal inference and is dependent on the accuracy and completeness of existing medical records, introducing the possibility of information and documentation bias. The single-center setting may restrict the generalizability of the findings, as surgical practices, peri-operative protocols, and patient demographics may differ across institutions and regions. The relatively modest sample size may limit statistical power, particularly for less frequent complications such as empyema, cardiac arrest, or thrombotic events, potentially underestimating rare but clinically significant outcomes. Although spirometric criteria were used to confirm COPD, detailed stratification according to disease severity, exacerbation history, and pharmacologic optimization was not comprehensively explored, which may influence postoperative outcomes. Potential confounding variables, such as intra-operative ventilatory strategies, fluid management, postoperative analgesic regimens, and adherence to pulmonary rehabilitation protocols, were not uniformly captured, limiting adjustment for all relevant peri-operative risk modifiers.

## Conclusions

Patients with COPD undergoing abdominal surgery represent a clinically vulnerable population characterized by advanced age, significant comorbid burden, and measurable peri-operative risk. Although spirometric parameters were relatively preserved in many patients, postoperative pulmonary and cardiac complications remained clinically relevant, contributing to extended critical care and hospital stay. The findings highlight that surgical risk in this group is multifactorial, influenced not only by baseline respiratory function but also by systemic disease status, operative exposure, and anesthetic duration. The overall mortality remained comparatively low, suggesting that careful peri-operative assessment and management can mitigate adverse outcomes. These results highlight the importance of structured pre-operative evaluation, optimized intra-operative strategies, and vigilant postoperative monitoring to improve surgical safety in patients with COPD.
